# A neuronal role of the Alanine-Serine-Cysteine-1 transporter (SLC7A10, Asc-1) for glycine inhibitory transmission and respiratory pattern

**DOI:** 10.1038/s41598-018-26868-6

**Published:** 2018-06-04

**Authors:** Guillaume Mesuret, Sepideh Khabbazzadeh, Anne M. Bischoff, Hazem Safory, Herman Wolosker, Swen Hülsmann

**Affiliations:** 1 Clinic for Anesthesiology, University Medical Center, Göttingen, Germany; 2 Center for Nanoscale Microscopy and Molecular Physiology of the Brain (CNMPB), Göttingen, Germany; 3 Department of Biochemistry, Rappaport Faculty of Medicine, Technion-Israel Institute of Technology, Haifa, 31096 Israel

## Abstract

The Alanine-Serine-Cysteine-1 transporter (SLC7A10, Asc-1) has been shown to play a role in synaptic availability of glycine although the exact mechanism remains unclear. We used electrophysiological recordings and biochemical experiments to investigate the role of Asc-1 transporter in glycinergic transmission in the brainstem respiratory network. Using both the Asc-1 substrate and transportable inhibitor D-isoleucine (D-Ile), and the non-transportable Asc-1 blocker Lu AE00527 (Lu), we found that D-Ile reduces glycinergic transmission and increases glycine release via hetero-exchange, whereas Lu has no acute effect on glycinergic synaptic transmission. Furthermore, D-Ile increases the frequency and reduces amplitude of the phrenic nerve activity in the arterially-perfused working heart brainstem preparation. These results suggest a role of Asc-1 in modulating presynaptic glycine levels that can impact on the respiratory network.

## Introduction

Glycinergic transmission plays an important role in the control of breathing. In the pre-Bötzinger complex (preBötC), which is crucial in the generation of breathing rhythm in mammals, glycinergic neurons represent a substantial population of inspiratory neurons^1–3^. While the role of glycinergic neurons in rhythmogenesis remains uncertain, glycinergic transmission has been found to strongly affect the amplitude and frequency of inspiratory and expiratory neuronal output^4,5^.

Transport of glycine in the brain is carried out mainly by the glial glycine transporter 1 (SLC6A9 or GlyT1), the neuronal glycine transporter 2 (SLC6A5 or GlyT2), which accumulates glycine in the cytosol of the presynapse, and the vesicular amino acid transporter (SLC32A1 or VIAAT)^6–9^. The latter transports both -aminobutyric acid (GABA) and glycine into synaptic vesicles^10–13^.

The Alanine-Serine-Cysteine-1 transporter (SLC7A10 or Asc-1) is a Na^+^-independent neutral amino acid antiporter distributed throughout the central nervous system^14–16^. Its primary role initially described in literature is the synaptic clearance of D-serine^17^. However, the transporter displays an high affinity for glycine in addition to serine, and recent studies have suggested that it is involved in the synaptic transport of glycine and glycinergic transmission in the brain^18–22^. Asc-1 knockout mice display low levels of glycine in the brain, decreased glycine inhibitory transmission^19,21^, and a hyperekplexia-like phenotype (similar to GlyT2 knockout mice) that can be rescued by L-serine or glycine application^21,23^.

Since the phenotype of Asc-1 knockout mice might be the consequence of the chronic brain depletion of glycine^21^, we aimed to address the consequences of acute interference with the Asc-1 transport using specific pharmacology. To test its role in glycinergic inhibitory transmission in the preBötC via whole cell recordings, we used two known Asc-1 inhibitors: (i) D-isoleucine (D-Ile), which is a transportable substrate for Asc-1 that uses the hetero-exchange (antiporter) activity of Asc-1^22^, and (ii) Lu AE00527 (Lu) as a none transportable antagonist, which has been tested to be specific for Asc-1 in the Asc-1 knockout mouse^19^. To assess the functional role of Asc-1 in the respiratory network, we additionally analyzed the phrenic nerve activity using the working heart brain preparation (WHBP).

## Results

### Glycinergic IPSCs in pre-Bötzinger complex

For our experiments, we used a double trangenic mouse, which allows identifying glycinergic neurons by the expression of EGFP under the control of GlyT2 promoter as well as GABAergic neurons by the expression of tdTomato under the GAD65-promoter^24^. In the preBötC, we could find many glycinergic cells expressing EGFP under the control of GlyT2 promoter (49,57 ± 6,63%), whereas a smaller number of cells expressed tdTomato (23,81 ± 6,81%) or both fluorophores (26,62 ± 2,72%).

**Figure 1:**
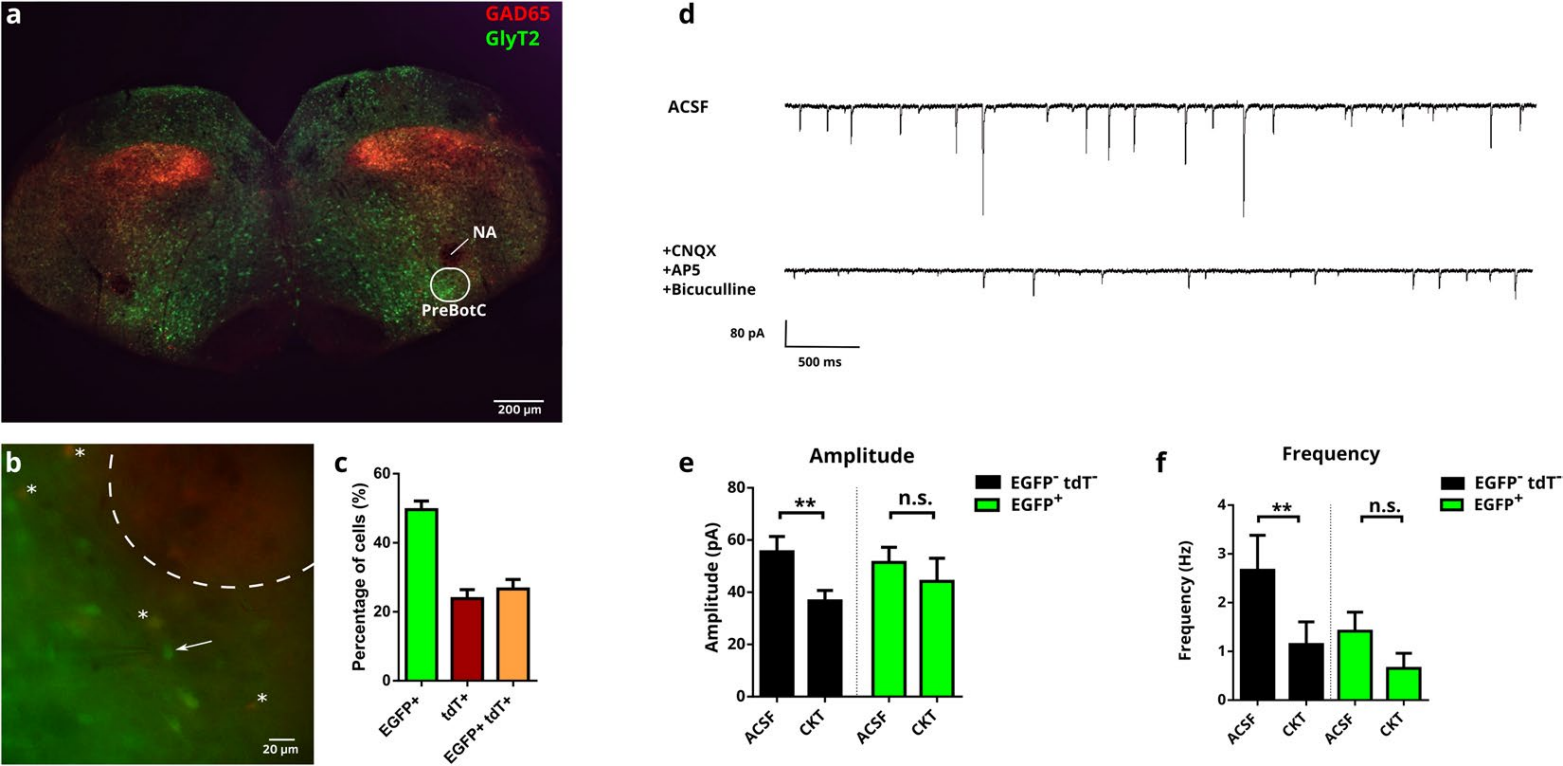
Glycinergic inhibitory transmission in the pre-Bötzinger complex. Coronal slice of the P6 mouse brainstem showing the preBötC (**a**). Fluorescent reporter proteins (EGFP for GlyT2, in green; tdTomato for GAD65, in red) allow identification of glycinergic and GABAergic neurons before approaching the cell with the patch pipette. NA: *nucleus ambiguus*. White circle demarks the preBötC. PreBötC at higher resolution (**b**) with the pipette patching a putative glycineric neuron (white arrow). Dual-fluorescent cells are indicated with an asterisk, and the NA with a dashed circle. The proportion of fluorescent cells shows a majority of GlyT2-EGFP cells over GABA-tdTomato or co-fluorescent cells in the preBötC (**c**). Representative traces (**d**) of spontaneous post synaptic currents (sPSC) in ACSF of a putative excitatory neuron from the preBötC (upper trace). To isolate glycinergic IPSCs, a cocktail of inhibitors (CKT) containing CNQX 20 μM, AP5 100 μM and bicuculline 20 μM was added to the ASCF (lower trace). Mean amplitude (**e**) and frequency (**f**) of sPSC and glycinergic IPSC recorded with the cocktail (CKT) are shown for glycinergic neurons (EGFP^+^, n = 17) and for non-glycinergic (EGFP^−^ tdT^−^), putative excitatory neurons (n = 16). The results are mean ± s.e.m; Wilcoxon’s matched-pairs t-test; **p > 0.01.

To assess glycinergic transmission in this area, we performed whole-cell voltage-clamp recordings on either EGFP-expressing glycinergic or non-fluorescent cells, which are presumably excitatory (Fig. 1). Glycinergic cells received less frequent spontaneous synaptic input compared to non-glycinergic cells (Fig. 1c). In ACSF, the frequency of spontaneous activity was 1.41 ± 0.72 Hz in glycinergic cells and 2.67 Hz ± 0.39 in non-glycinergic cells (p < 0.05). The addition of D-AP5, CNQX and bicuculline to isolate glycinergic IPSCs significantly reduced the amplitude and frequency of spontaneous post-synaptic currents in non-glycinergic cells. In glycinergic cells, although the mean frequency showed a 2-fold reduction, the effect is not significant, presumably because glycinergic cells receive less overall input and some hardly show any spontaneous activity. For the following analysis of the role of Asc-1 on glycinergic transmission, we therefore decided to use only glycinergic IPSCs that were recorded from non-glycinergic neurons.

### D-Isoleucine but not Lu AE00527 reduces glycinergic currents

**Figure 2:**
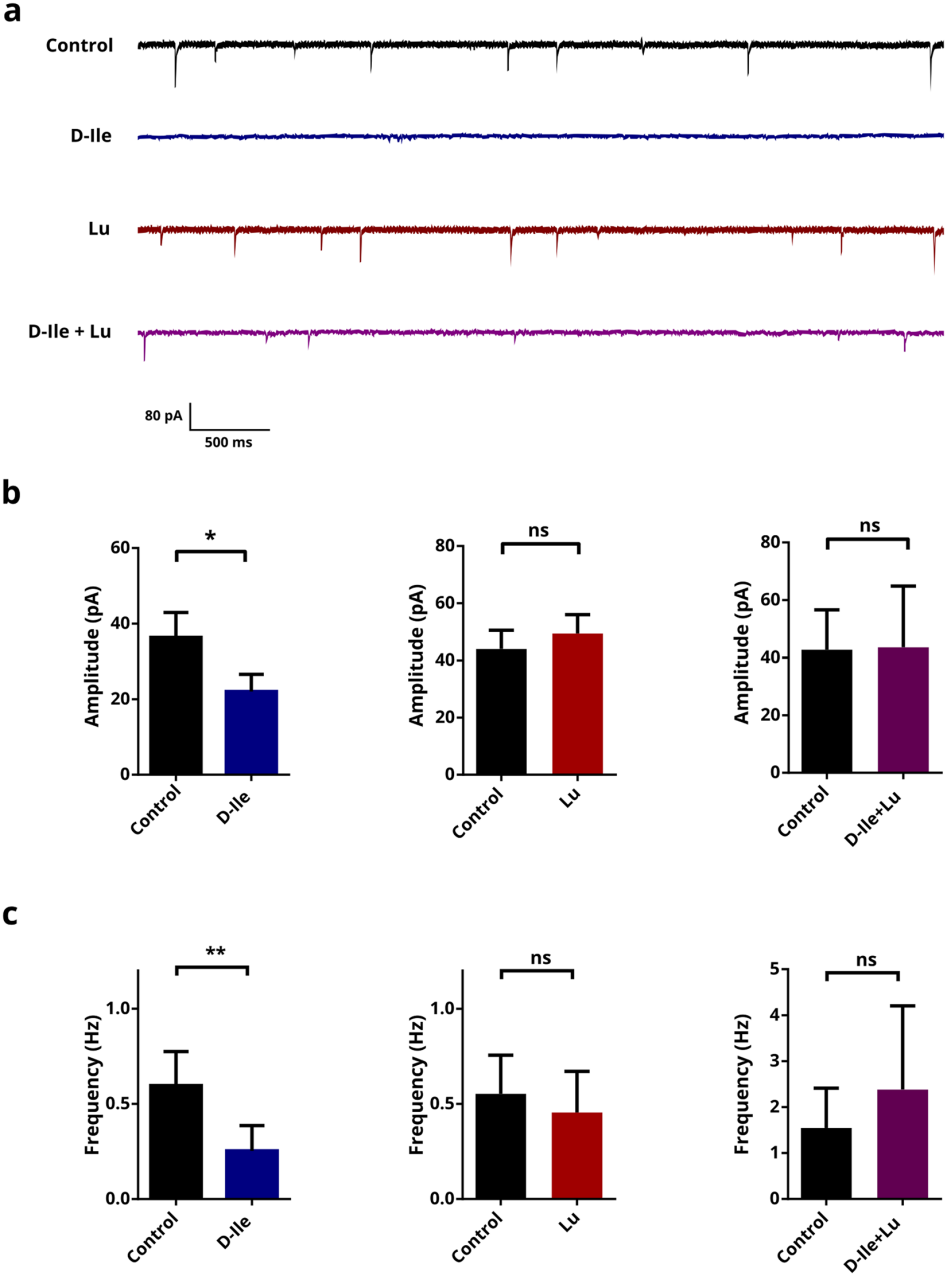
Effects of Asc-1 inhibitors on IPSCs in brainstem slices. (**a**) Representative traces of glycinergic IPSCs in control conditions (in presence of CNQX, AP5 and bicuculline) and with D-isoleucine (D-Ile, 1 mM), Lu AE00527 (Lu; 10 µM), or application of D-Ile and Lu together. Mean amplitude (**b**) and frequency (**c**) of glycinergic currents before and after application of 1 mM D-Ile, 10 μM Lu, or a combination of Lu and D-Ile. The results are mean ± s.e.m. from 9, 6 and 6 slices. *^,^**Different from control at p < 0.05 and 0.01, respectively. Wilcoxon’s matched-pairs t-test.

In a first set of experiments, we used D-Isoleucine (D-Ile), a selective-transportable inhibitor of the transporter Asc-1^19^, to investigate the role of Asc-1 on the glycinergic IPSCs (Fig. 2). In putative excitatory (non-fluorescent) neurons of the preBötC, D-Ile reduced significantly both the amplitude and frequency of IPSCs. The amplitude was reduced from 36.33 ± 6.62 pA to 22.04 ± 4.54 pA (n = 9, p < 0.05) and the frequency was reduced from 0.39 ± 0.10 to 0.20 ± 0.07 Hz with the addition of 1 mM of D-Ile (p < 0.01). However, the use of Lu AE00527 (Lu), which is a non-transportable blocker of Asc-1, did not have any significant effect by itself. The amplitude was 43.97 ± 6.55 and 49.38 ± 6.64 pA and the frequency was 0.55 ± 0.20 and 0.46 ± 0.22 Hz (n = 6, n.s.).

Asc-1 is known to work as an antiporter for neutral amino acids^14^. D-Ile was shown to activate Asc-1 hetero-exchange in primary neuronal culture slices, whereby D-Ile influx though the transporter is coupled to the release of intracellular Asc-1 substrates (e.g. glycine or D-serine)^22^. On the other hand, Lu AE 00527 was shown to block the transporter activity, without eliciting hetero-exchange^19^. We therefore tested the hypothesis that D-Ile decreases the frequency and amplitude of glycinergic IPSCs by depleting the neurons from glycine by eliciting a D-Ile_(in)_/glycine_(out)_ exchange through Asc-1. If this is the case, Lu AE00527 should block D-Ile effects. In agreement, we found that Lu AE00527 abolishes the effect of D-Ile. The amplitude was 42.76 ± 13.82 and 43.58 ± 21.30 pA and the frequency 1.55 ± 0.87 Hz to 2.38 ± 1.82 Hz (n = 6, n.s.) for the control and combination of D-Ile and Lu AE00527, respectively.

### D-Isoleucine promotes glycine release in the brainstem

**Figure 3:**
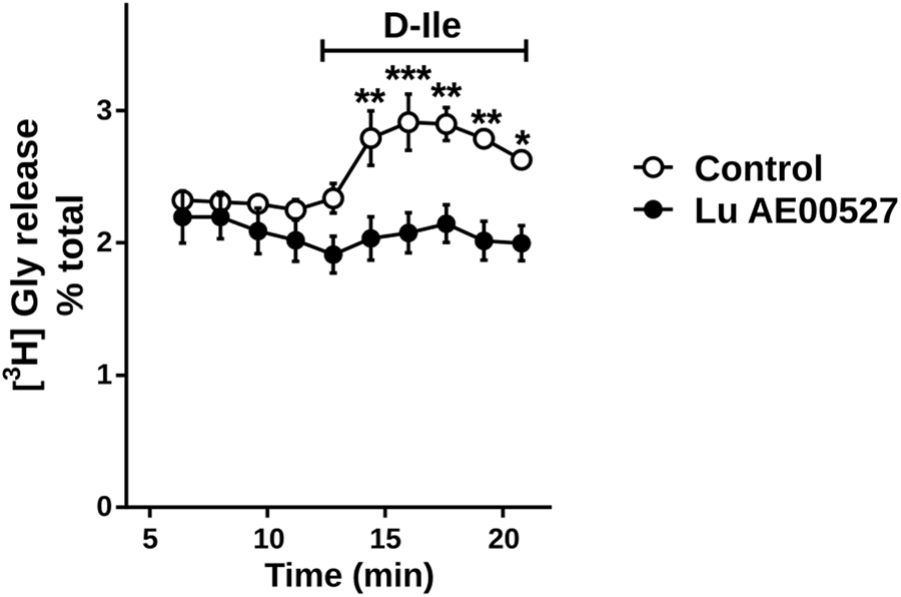
Effects of an Asc-1 inhibitor on glycine release from brainstem slices. Addition of D-Isoleucine (D-Ile, 1 mM) promotes an increase in the release rate of preloaded ^3^H-glycine. This release was mostly blocked by inclusion of 10 μM Lu AE00527 five minutes prior to the application of D-Ile. The results are mean ± s.e.m. of 5 experiments. Two-way Repeated Measures ANOVA with Bonferroni’s multiple comparison test: *^,^**^,^***different from control at p < 0.05, 0.01, and 0.001, respectively.

In order to determine the effects of the two inhibitors on glycine release in the brainstem, we applied D-Ile either in the absence or in the presence of Lu AE00527 on brainstem slices while monitoring glycine release (Fig. 3). Perfusions of the slices with D-Ile increased the fractional rate of glycine release, compatible with D-Ile_(in)_/glycine_(out)_ exchange. D-Ile effect was strongly reduced when D-Ile was applied in the presence of Lu AE00527. The data support the notion that D-Ile promotes presynaptic glycine depletion by activating Asc-1 antiporter activity, while Lu blocks all transport modes operated by Asc-1 and prevents the glycine leakage via Asc-1.

### Effects on phrenic nerve activity

**Figure 4:**
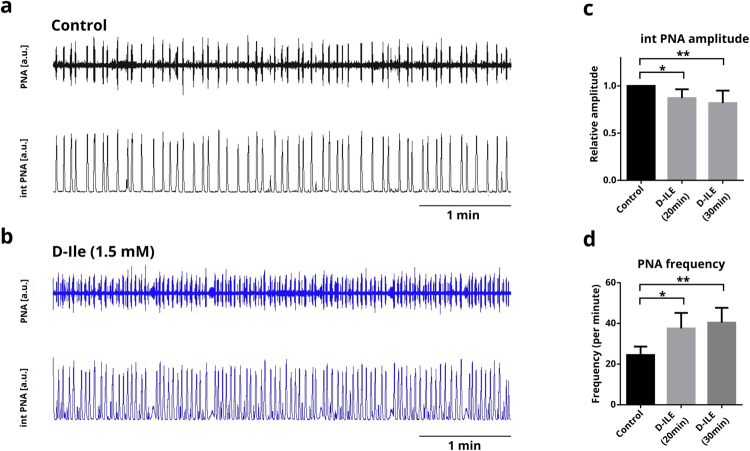
Recordings of phrenic nerve activity (PNA) in the working heart brain preparation in mouse. (**a**,**b**) raw phrenic nerve activity (PNA) is shown in the upper traces and integral (int. PNA) in the lower traces. (**a**) Control recordings in aCSF, (**b**) recordings after 20 min of D-Ile (1.5 mM). (**c**) D-Ile decreased the amplitude of the int. PNA peaks 20 min and 30 min after application and increased the frequency of PNA (n = 10). Data are mean ± S.E.M (n = 10). Holm-Sidak’s multiple comparison test (one way ANOVA).

In order to assess the effect of D-Ile on the respiratory network, we recorded the phrenic nerve activity (PNA) in an arterially-perfused working heart brainstem preparation (WHBP) (Fig. 4). D-Ile decreased the amplitude of the peaks to 0.87 ± 0.03 and 0.82 ± 0.04, 20 min and 30 min after application, respectively; while the frequency of PNA was increased from 24.46 ± 4.18 to 37.53 ± 7.64 after 20 min, and to 40.36 ± 7.30 after 30 min (Fig. 4, n = 10). In a preliminary set of data (Supplementary Figure, n = 3) we tested whether the effect of D-Ile can be blocked by the application of Lu AE00527 prior to the application of D-Ile. In the presence of Lu AE00527, D-Ile-did not increase PNA-frequency. There was rather a tendency to slower network output.

## Discussion

In the present study, we used co-fluorescent mice to differentiate glycinergic cells from GABAergic and glutamatergic cells. We observed that glycinergic cells received less overall input. We showed that the transportable Asc-1 inhibitor D-Ile reduces the glycinergic post synaptic currents of putative excitatory neurons, while Lu AE00527 did not. Our experiments show that D-Ile promotes glycine release from brainstem slices, compatible with the activation of the Asc-1 hetero-exchange mechanism^25^. This suggests that glycine depletion is likely the result of excessive hetero-exchange between D-Ile and glycine - and possibly serine as well.

Asc-1 has been reported to be expressed in neurons^16,26,27^. However, Asc-1 was also found to be expressed in astrocytes in the brainstem^28^ and the spinal cord^20^. Whether or not Asc-1 is quantitatively more expressed in astrocytes or in neurons still remains to be evaluated. However, our present results suggest that the effects of D-Ile on the inhibitory glycinergic transmission are compatible with a functional role of Asc-1 in neurons. The decrease in the frequency and amplitude of the glycinergic IPSCs cannot be explained by an expression of Asc-1 only in astroglia as proposed by Ehmsen and coworkers^20^. In case Asc-1 only facilitates glycine release from astroglia by hetero-exchange as proposed by Ehmsen *et al*., one would expect an increase in glycinergic tone promoted by D-Ile due to higher glycine release from astrocytes, while the addition of Lu AE00527 would be predicted to have the opposite effects. Our experiments suggest that the acute effect of D-Ile in the neuronal pool of Asc-1 surpasses its effects on astrocytic Asc-1, as D-Ile promotes a large decrease in frequency and amplitude of IPSCs, attributable to depletion of pre-synaptic glycine.

Although Lu AE00527 counteracted the effects of D-Ile, its lack of effect on glycinergic transmission when present alone was somewhat surprising and discrepant from the Asc1 knockout data^20^. This result indicates nonetheless that the basal activity of Asc-1 does not acutely regulate the glycinergic neurotransmission under our recording conditions. Therefore, the decrease in the levels of brain glycine and the impairment of the glycinergic inhibitory neurotransmission in Asc-1-KO mice is more likely related to the combination of reduced biosynthesis of glycine from serine^29^ or by decreased glycine export from glia^20^. Together this would suggest that the hyperekplexia phenotype due to glycinergic impairment in Asc-1-KO mice is related to a chronic alteration in glycine metabolism, rather than a role of Asc-1 as an acute regulator of presynaptic glycine.

**Figure 5:**
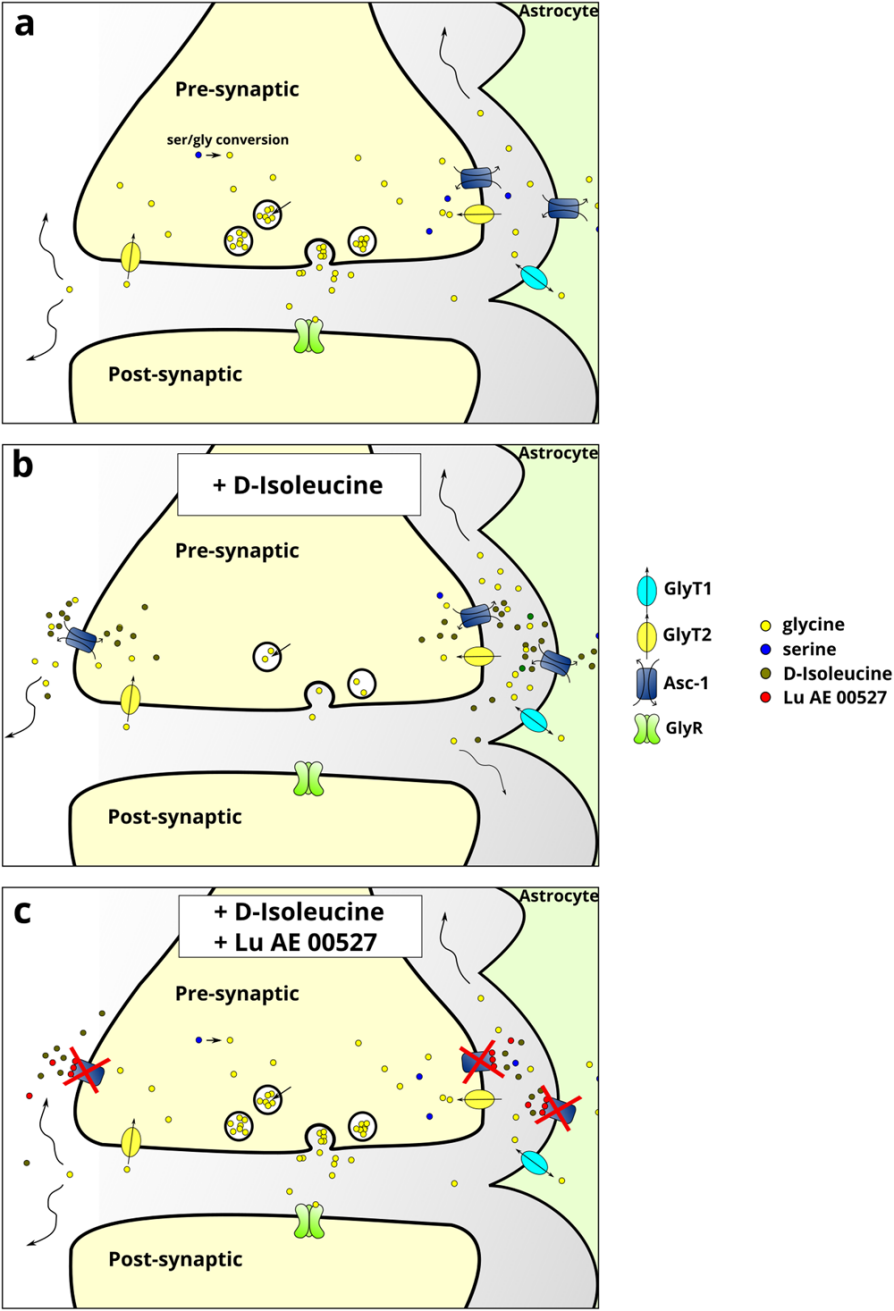
Schematic illustration representing the effect of Asc-1 inhibitors on glycinergic transmission. Asc-1 acts as a shuttle for glycine and serine in neurons and astrocytes depending on their gradients (**a**). D-Ile enhances the antiporter activity of Asc-1, thus depleting the neurons from serine and glycine (**b**). The overall effect of D-Ile in the neuronal pool of Asc-1 should surpass its effects on astrocytic Asc-1, as D-Ile promotes a large decrease in frequency and amplitude of IPSCs. Lu blocks the activity of Asc-1 completely (**c**). Glycine can still be recaptured by GlyT2 and the transport of serine by other transporters should be unchanged. However, once the Asc-1 is blocked by the non-transportable inhibitor (Lu), it becomes insensitive to D-Ile, which cannot deplete glycine from the presynaptic terminals, as Lu blocks all transport modes of Asc-1.

Maucler *et al*.^25^ have demonstrated that Asc-1 can release D-serine via hetero-exchange *in vivo*. Thus, it is likely that Asc-1 can slowly equilibrate the serine and glycine pools between neurons and astrocytes in glycinergic synapses, acting as a shuttle between them via its antiporter/heteroexchanger activity, until equilibrium between the amino acids is reached (Fig. 5).

In this framework, we propose that in the brainstem Asc-1 might use the high concentration of glycine that is set by GlyT2 at glycinergic synapses^30^ to promote the uptake of D-serine and other substrates. The preferred direction of glycine flux via Asc-1 in neurons and in astrocytes and the direction of transport of serine and other amino acids under physiological conditions remains to be elucidated.

Our data are consistent with the vast literature indicating the importance of glycine for respiratory control. However, since D-Ile not only impairs phasic inhibition but also effects tonic inhibition, each having opposite results on the network^31,32^, the net effect is difficult to predict. Moreover, systemic application of D-Ile causes depletion of glycinergic neurons not only in pre-Bötzinger neurons, but also in other respiratory centers as well as in the cervical spinal cord. Additionally, unlike in the slice preparation, alterations in the extracellular glycine and serine levels probably affect not only glycinergic inhibition but also excitatory neurotransmission. Nonetheless, the D-Ile-mediated increase of phrenic nerve rate observed in the working heart brain-preparation is in line with the effect of systematic blockade of glycinergic synaptic inhibition in the WHBP with strychnine^33,34^. Thus, depletion of glycine by acute addition of D-Ile reduces the net inhibition in the respiratory network through enhancement of Asc-1 hetero-exchange. The data support the notion that a significant reduction of glycinergic inhibition is not able to impair the central rhythm generating network but certainly alters pattern formation^35^.

## Methods

### Animals

Animals were bred in the central animal facility of the University Medical Center of the Georg-August University Göttingen and treated in accordance with the German Protection of Animals Act (TierSchG) and with the guidelines for the welfare of experimental animals issued by the European Communities Council Directive 2010/63/EU. Procedures (§ 4 Abs. 3 TierSchG) were approved and registered (T12/11) by the animal welfare office and commission of the University Medical Center Gottingen. The double transgenic mouse line used allows the visualisation of inhibitory neurons and the distinction of glycinergic and GABAergic cells. This line was generated by crossing GlyT2-EGFP mice expressing the green fluorescent protein EGFP under the GlyT2 promotor in glycinergic neurons^36^ with GAD65-tdTomato mice expressing the red fluorescent protein tdTomato under the control of the GAD65 promotor in GABAergic neurons^24^.

### Acute brainstem slice preparation

Mice at postnatal day 2 to 10 were anaesthetised with isoflurance, killed by decapitation, and their brains were removed. The brainstem was isolated in ice-cold oxygenated artificial cerebrospinal fluid (ACSF) containing 118 mM NaCl, 3 mM KCl, 1.5 mM CaCl_2_, 1 mM MgCl_2_, 1 mM NaH_2_PO_4_, 25 mM NaHCO_3_, and 30 mM D-glucose. The brainstem was then fixed on an agar block and sectioned on a vibratome (VT1200S, Leica). Coronal slices (200 μm) were collected at the levels of the fourth ventricle and kept in oxygenated ACSF at room temperature for at least 20 min before the experiment in order to allow slice recovery. Recordings of pre-Bötzinger complex neurons were performed at the level of the inferior olive, and ventral to the Nucleus ambiguus, which was clearly recognizable by the lack of EGFP fluorescence in a round pattern (Fig. 1a).

### Electrophysiology

Brainstem slice were transferred to the recording chamber perfused with oxygenated ACSF. The temperature of the bath was kept at 28 °C with a SC100 circulating bath controller (Thermo Scientific). Cells were observed under an epifluorescence microscope Examiner Z1 (Zeiss), illuminated with SOLA SE light engine (Lumencor), and captured with a Sensicam CCD camera (PCO Imaging) controlled by Imaging Workbench software (Indec Biosystems). Patch electrodes were made from glass capillaries using a horizontal pipette puller (Zeitz Intrumente), and were filled with an intracellular solution containing 130 mM KCl, 1 mM CaCl_2_, 2 mM MgCl_2_, 2 mM Na_2_ATP, 10 mM HEPES, 10 mM EGTA. After obtaining a giga-ohm seal, the membrane was ruptured and the membrane potential was set to −70 mV using a Multi-Clamp 700 A patch clamp amplifier. Whole-cell patch-clamp recordings were conducted in voltage clamp mode and acquired with Digidata 1322 A (Axon instrument). To isolate glycinergic spontaneous inhibitory post-synaptic currents, a cocktail of 50 μM 2-amino-5-phosphopentanoate (D-AP5), 20 μM 6-cyano-7-nitroquinoxaline-2,3-dione (CNQX) and 20 μM Bicuculline (Sigma-Aldrich) was added to the bath. D-isoleucine 1 mM (D-Ile) or 10 μM Lu AE00527 (Lu) was added to the cocktail 20 min before recording. In case both were applied, Lu was added 1 min before D-Ile in order to block the transporter prior to D-Ile effects. Lu AE00527 was provided by H. Lundbeck A/S (Denmark).

### Image acquisition and cell counting

Visualisation of fluorescent protein expression in slice was either performed directly at the setup described above or using a confocal laser-scanning microscope (LSM, 510 META, Axiovert 200, Zeiss, Oberkochen, Germany). At the LSM, EGFP emission (EM) was detected via a 505–530 nm bandpass-filter (BP) with 488 nm for excitation (EX) and tdTomato EM was recorded through a 560 nm longpass filter (543 nm for EX). At the electrophysiology setup, fluorescence could be recorded at the side port of the Examiner Z1 via a color camera (DFK41BU02, The Imaging Source Europe GmbH). EGFP was detected using a filter set (Zeiss, Filter set 38) consisting of a BP 450–490 nm for EX, a beam splitter (BS) 495 nm, and an EM filter BP 500–550 nm. For the separation of tdTomato-fluorescence a Texas red filter set (F20–309; AHF analysentechnik AG) was used (EX: BP 580–604 nm; BS 615 nm; EM BP 625–725 nm). For cell counting, composites of the EGFP and tdTomato images were generated in ImageJ (https://fiji.sc/) and assessed with the *cell counter* plugin by two investigators. Additionally, a dualband filter set (F56-019, AHF analysentechnik AG) was used to visualise EGFP and tdTomato at once.

### Glycine release from acute slices

Ten-week old C57Bl/6 mice were anesthetised with isoflurane and killed by decapitation in accordance with the Committee for the Supervision of Animal Experiments (Technion–Israel Institute of Technology). The brainstem was dissected and chopped into strips measuring 400 μm by 400 μm using a McIlwain tissue chopper. Brainstem slices were loaded with 2 μM [³H] glycine by 20 min incubation in oxygenated Krebs-HEPES buffer (MKB) (in mM: 127 NaCl, 1.3 NaH_2_PO_4_, 15 HEPES, 10 D-glucose, 1 MgCl_2_, 5 KCl, and 2.5 CaCl_2_, pH 7.4). After 3 washes with MKB, the slices were transferred to the Suprafusion 1000 (SF-6) apparatus (Brandel) and equilibrated by a 20 min perfusion with oxygenated MKB buffer at a flow rate of 0.6 ml/min at 37 °C. After equilibration in perfusion medium (either control Krebs buffer or Krebs buffer plus 10 μM Lu AE00527), 1 mM D-Ile was added to the perfusate, which was collected at 1.6 min intervals, and the radioactivity was monitored by scintillation counting using Ultima Gold scintillation solution (Perkin-Elmer). Under our experimental conditions, little metabolism of glycine was observed during the perfusion^22^.

### Working heart brain preparation

The working heart-brain stem preparation (WHBP)^37^ was prepared as described elsewhere^34,38^. Briefly, mice were anesthetised deeply with isoflurane until apnea occurred and the nociceptive withdrawal reflex was eliminated. Animals were decerebrated at the pre-collicular level and cerebellectomised, bisected below the diaphragm, and the skin, lungs and diaphragm were removed; the descending aorta and thoracic phrenic nerve (PN) were isolated and cut distally. The preparation was placed in a recording chamber and perfused retrogradely at 10–20 ml/min with ACSF by a peristaltic roller pump (Watson Marlow). The perfusion solution consisted of carbogenated (95% O_2_, 5–8% CO_2_) ACSF at 32 °C containing an oncotic agent (Ficoll 1.25%; Sigma). The second lumen of the catheter was used to monitor perfusion pressure. The baseline flow of the perfusate was set to 16–20 mL/min and adjusted to observe uniform readily quantitatible phrenic nerve discharges under control conditions. The composition of the ACSF was (in mM): 125 NaCl; 25 NaHCO_3_; 2.5 CaCl_2_; 1.25 MgSO_4_; 1.25 KH_2_PO_4_; 5 KCl; 10 glucose, osmolarity was 300 ± 10 mOsm/L. Phrenic nerve activity (PNA) was recorded using custom-made glass suction electrodes. PNA signals were amplified 12 500 times, band-pass filtered (0.25–2 kHz) and integrated (time constant, 0.3–0.6 ms) using a custom-made amplifier (electronic workshop of the department of physiology, Göttingen). Signals were digitized by a PowerLab 8/30.

### Data Handling

Electrophysiological data were exported as.ABF files and analyzed with Minianalysis 6.0 (Synaptosoft Inc.). Data are given as as means ± standard error mean. Statistical tests were performed in GraphPad Prism software, and differences were considered statistically significant if p < 0.05. For the WHBP experiments, PNA signals were analysed using LabChart software (ADInstruments). The averaged amplitude of the integrated PNA signal during a period of one minute before the application of D-ILE was calculated for each experiment. Alterations induced by D-ILE were calculated from a period of 1 min and expressed as the fraction of the baseline activity (relative PNA). The datasets generated during or analysed during the current study are available from the corresponding author on reasonable request.

## Electronic supplementary material

Supplementary Information
