# Supplementary material for: A neuronal role of the Alanine-Serine-Cysteine-1 transporter (SLC7A10, Asc-1) for glycine inhibitory transmission and respiratory pattern
Source: Sci Rep. 2018 Jun 4;8:8536. doi: 10.1038/s41598-018-26868-6 (PMC5986860; doi:10.1038/s41598-018-26868-6)
Supplement: Supplementary file 1 — Supplementary Information [file 41598_2018_26868_MOESM1_ESM.pdf]

Supplementary Information

A neuronal role of the Alanine-Serine-Cysteine-1 transporter (SLC7A10, Asc-1) for glycine inhibitory transmission and respiratory pattern

Guillaume Mesuret[1,2], Sepideh Khabbazzadeh[1], Anne M. Bischoff[1], Hazem Safory[3], Herman Wolosker[3], Swen Hülsmann*[1,2]

[1] Clinic for Anesthesiology, University Medical Center, Göttingen, Germany.

[2] Center for Nanoscale Microscopy and Molecular Physiology of the Brain (CNMPB), Göttingen, Germany.

[3] Department of Biochemistry, Rappaport Faculty of Medicine and Research Institute, Technion-Israel Institute of Technology, Haifa 31096, Israel.

* Corresponding author (email : shuelsm2@uni-goettingen.de)

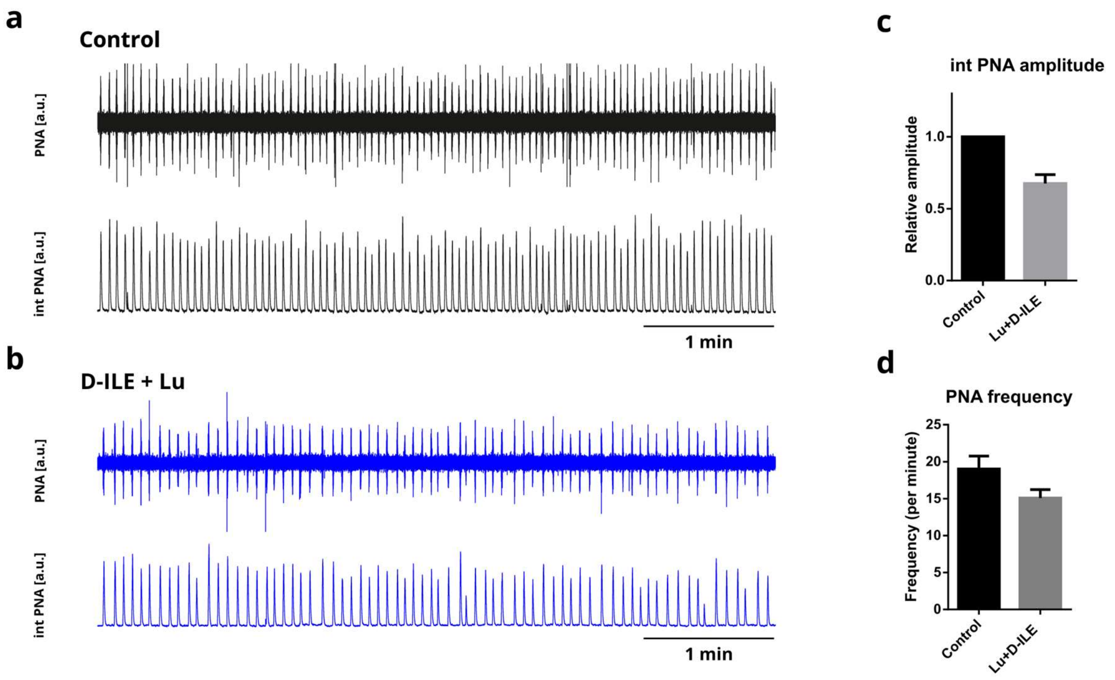

Supplementary Figure S1 : Lu AE00527 diminishes the effect of D-Ile on phrenic nerve activity (PNA) in the working heart brain preparation. (a,b) Recordings of phrenic nerve activity PNA, upper trace) and integral (int. PNA, lower trace) in the working heart brain preparation in mouse. **(a)** Control recordings in ACSF, **(b)** recordings after 20 min of D-Ile (1mM), which was applied 5 min after Lu AE00527 (10 µM). D-Ile does not produce an increases of the PNA frequency after application of Lu AE0257. **(c,d)** Bar charts showing mean ± s.e.m for the amplitude **(c)** of the integrated phrenic nerve activity (int PNA) normalized to control, and the frequency **(d)** of phrenic nerve activity (per minute). Paired t-test; n.s. (n = 3).
